# Transnasal Humidified Rapid Insufflation Ventilatory Exchange in children requiring emergent intubation (Kids THRIVE): a statistical analysis plan for a randomised controlled trial

**DOI:** 10.1186/s13063-023-07330-z

**Published:** 2023-05-31

**Authors:** Shane George, Kristen Gibbons, Tara Williams, Susan Humphreys, Ben Gelbart, Renate Le Marsney, Simon Craig, David Tingay, Arjun Chavan, Andreas Schibler, for the Kids THRIVE Investigators, Joanna Cronin, Kylie Pearson, Katie Rasmussen, Jason Acworth, Leah Hickey, Carmel Delzoppo, Elizabeth Perkins, Felix Oberender, Jessica Waghorn, Courtney McCahill, Nitesh Singhal, Gail Harper, Subodh Ganu, Georiga Letton, Simon Erikson, Hannah Thomson, Luregn J. Schlapbach, Juerg Burren, Elisa Zimmerman, Anusha Ganeshalingham, Stuart Dalziel, Clarie Sherring, Paediatric Research in Emergency Departments International Collaborative (PREDICT), the Australia, New Zealand Intensive Care Society Paediatric Study Group (ANZICS PSG)

**Affiliations:** 1 Departments of Emergency Medicine, Children’s Critical Care, Gold Coast University Hospital, 1 Hospital Boulevard, Southport, QLD Australia; 2 Child Health Research Centre, The University of Queensland, South Brisbane, Australia; 3 School of Medicine and Menzies Health Institute Queensland, Griffith University, Gold Coast Campus, Southport, Australia; 4 Division of Critical Care Medicine, Queensland Children’s Hospital, South Brisbane, Australia; 5 Paediatric Intensive Care Unit, Royal Children’s Hospital, Melbourne, VIC Australia; 6 Department of Paediatrics, University of Melbourne, Melbourne, VIC Australia; 7 Clinical Sciences, Murdoch Children’s Research Institute, Melbourne, VIC Australia; 8 Paediatric Emergency Department, Monash Medical Centre, Monash Emergency Research Collaborative, Monash Health, Clayton, VIC Australia; 9 Department of Paediatrics, Monash University, Melbourne, VIC Australia; 10 Department of Neonatology, The Royal Children’s Hospital, Melbourne, VIC Australia; 11 Neonatal Research, Murdoch Children’s Research Institute, Melbourne, VIC Australia; 12 Paediatric Intensive Care Unit, Townsville University Hospital, Townsville, Australia; 13 St Andrew’s War Memorial Hospital, Brisbane, Australia; 14 Critical Care Research Group, St Andrew’s War Memorial Hospital, Brisbane, Australia; 15 Wesley Medical Research, Auchenflower, Queensland Australia

## Abstract

The placement of an endotracheal tube for children with acute or critical illness is a low-frequency and high-risk procedure, associated with high rates of first-attempt failure and adverse events, including hypoxaemia. To reduce the frequency of these adverse events, the provision of oxygen to the patient during the apnoeic phase of intubation has been proposed as a method to prolong the time available for the operator to insert the endotracheal tube, prior to the onset of hypoxaemia. However, there are limited data from randomised controlled trials to validate the efficacy of this technique in children. The technique known as transnasal humidified rapid insufflation ventilatory exchange (THRIVE) uses high oxygen flow rates (approximately 2 L/kg/min) delivered through nasal cannulae during apnoea. It has been shown to at least double the amount of time available for safe intubation in healthy children undergoing elective surgery. The technique and its application in real time have not previously been studied in acutely ill or injured children presenting to the emergency department or admitted to an intensive care unit. The Kids THRIVE trial is a multicentre, international, randomised controlled trial (RCT) in children less than 16 years old undergoing emergent intubation in either the intensive care unit or emergency department of participating hospitals. Participants will be randomised to receive either the THRIVE intervention or standard care (no apnoeic oxygenation) during their intubation. The primary objective of the trial is to determine if the use of THRIVE reduces the frequency of oxygen desaturation and increases the frequency of first-attempt success without hypoxaemia in emergent intubation of children compared with standard practice. The secondary objectives of the study are to assess the impact of the use of THRIVE on the rate of adverse events, length of mechanical ventilation and length of stay in intensive care. In this paper, we describe the detailed statistical analysis plan as an update of the previously published protocol.

**Supplementary Information:**

The online version contains supplementary material available at 10.1186/s13063-023-07330-z.

## Introduction

### Background and rationale

The placement of an endotracheal tube for children with acute or critical illness is a low-frequency and high-risk procedure, associated with high rates of first-attempt failure and adverse events, including hypoxaemia [1]. To reduce the frequency of these adverse events, the provision of oxygen to the patient during the apnoeic phase of intubation has been proposed as a method to prolong the time available for the operator to insert the endotracheal tube, prior to the onset of hypoxaemia [2]. However, there are limited data from randomised controlled trials to validate the efficacy of this technique in children.

The technique known as transnasal humidified rapid insufflation ventilatory exchange (THRIVE) uses high oxygen flow rates (approximately 2 L/kg/min) delivered through nasal cannulae during apnoea. It has been shown to at least double the amount of time available for safe intubation in healthy children undergoing elective surgery [3]. The technique and its application in real time have not previously been studied in acutely ill or injured children presenting to the emergency department or admitted to an intensive care unit.

### Intervention

A detailed description of the intervention is included in the previously published protocol paper [4]. In brief, eligible participants will be randomised to receive either standard care (control) or THRIVE during the apnoeic period of endotracheal intubation.

Participants randomised to standard care will be intubated according to site-specific guidelines or clinician preference, with no provision of apnoeic oxygen during the intubation. Those randomised to the THRIVE intervention will be administered high-flow oxygen through nasal cannula at a flow rate of approximately 2 L/kg/min. The intervention will be applied immediately after the mask or device used for preoxygenation is removed and before the laryngoscope is introduced into the mouth.

The intubation procedure will proceed according to standard hospital procedures and clinician preference, with study data continuously collected during the procedure.

### Objectives

The primary objective of the trial is to determine if the use of THRIVE reduces the frequency of oxygen desaturation and increases the frequency of first-attempt success without hypoxaemia in emergent intubation of children compared with standard practice. The secondary objectives of the study are to assess the impact of the use of THRIVE on the rate of adverse events, length of mechanical ventilation and length of stay in intensive care.

## Study methods

### Trial design

The Kids THRIVE trial is a multicentre, international, randomised controlled trial (RCT) in children less than 16 years old undergoing emergent intubation in either the intensive care unit or emergency department of participating hospitals. Participants will be randomised to receive either the THRIVE intervention or standard care (no apnoeic oxygenation) during their intubation. Further details can be found in the previously published protocol [4].

### Sample size

A conservative estimate of the primary outcome of hypoxaemia is set at 16%, following an unpublished 2016 audit of data from the Queensland Children’s Hospital, which we found was comparable to a published report [1]. Assuming a clinically relevant effect size of a 50% reduction in the proportion of desaturation events from 16 to 8%, along with a type I error of 0.025 (adjusting for two primary outcomes using Bonferroni’s correction) and power of 90%, 816 intubation encounters in total are required. Successful first-attempt intubation, the second primary outcome, assumes an absolute increase from 60 to 80%, requiring 258 intubation encounters. Allowing for 15% attrition, the total sample size that will satisfy both primary outcome measures is 960 intubation encounters. An attrition rate of 15% was selected based on the assumption that a proportion of patients enrolled will have a clinical deterioration, or other clinical events, during a high-stress procedure leading to the inability to deliver the intervention or collect the outcome data required for the study.

### Randomisation

A computer-generated randomisation sequence using block randomisation (block size = 10) with a 1:1 ratio and stratification by site, age (< 1 year, 1–7 years and > 7 years of age), then level of the intended first operator seniority (junior and senior medical officer) was developed. Randomisation is conducted using sequential, numbered, sealed, opaque envelopes, provided to each site. The use of a fixed block size is unlikely to reduce the allocation concealment as randomisation occurs by any member of the large clinical team, including when the research staff are not present outside of usual business hours. Thus, any person selecting the envelope is unlikely to be aware of the previous allocations in the block. After a patient has been screened and confirmed as eligible and the operator for the intubation has been determined, the enrolling clinician selects the next envelope to reveal the study randomisation for that intubation encounter.

### Interim analyses

An independent Data and Safety Monitoring Board (DSMB) has been convened for this study.

Interim analyses were pre-defined to assess the progress and safety of the trial after the primary outcome was known for 100 and 200 children. At the DSMB establishment meeting, the DSMB requested a modification to the interim analysis schedule to include a safety review at 100 patients, a safety and efficacy analysis at 300 patients and removal of the 200-patient review point. This was requested as the study was powered for one of the primary outcomes once outcomes were available for 300 children. The request was discussed and agreed upon by the trial steering committee and incorporated into the updated protocol. While no formal stopping rule was used, the DSMB could recommend ceasing the trial if there is a statistically significant difference (*p* < 0.001) in the primary outcome between the treatment groups overall or within pre-specified age subgroups, or in the case of serious adverse events. Cessation of the trial could also be recommended if there is equipment failure or recall, or if other evidence becomes available that would make continuing the trial unethical.

The blinded outcome information was presented to the DSMB by a pseudo-labelled treatment arm. No unblinding of the data was requested by the DSMB. The DSMB recommended the continuation of the trial with no protocol changes following a review of data and adverse events at both interim analyses.

### Timing of final analysis

The final analysis will be conducted after data entry and monitoring have been completed and the database is cleaned and closed.

### Data sources

Four data sources are being used for data collection:

- Clinical record: data from the patient’s hospital medical record including demographics, diagnosis, admission and intubation information.
- Paper case report form (CRF): source data including patient observations and intubation-specific details will be entered directly onto a paper CRF by a researcher or clinician during the intubation.
- Video of the intubation attempt/s: a video recording device has been provided to each site and placed in a location to obtain a full view of the intubating clinician and child’s face (additional information on this aspect previously published [4]). Where there is a discrepancy between manually recorded data and video data, the video-collected data will be used for analysis. Videos are stored locally and then uploaded to The University of Queensland (UQ) Research Data Manager (RDM).
- The Australian and New Zealand Paediatric Intensive Care Registry (ANZPICR): the ANZPICR identifier is collected for each patient admitted to a paediatric intensive care unit. These identifiers are provided to the ANZPICR who subsequently provide an extract containing information relating to diagnosis, demographic characteristics, start and stop times of mechanical ventilation episodes and outcomes.

Data from all four sources is collected and entered into the purpose-built study database developed in REDCap and hosted by UQ [5, 6], either by direct entry, paper record upload or import.

### Data monitoring

Data monitoring is being undertaken throughout the trial, based on a data monitoring plan devised by the study team. The data monitoring plan was developed in accordance with the ICH E6 (R2) Good Clinical Practice Guideline [7] and reflects the current best practice for data monitoring practices in investigator-initiated trials. Briefly, the data monitoring plan includes the following components:

- Data verification on all screening data items (i.e. inclusion and exclusion criteria) for every enrolled patient (utilising the clinical record)
- Data verification on the stratification used for randomisation and consent data items for every enrolled patient (utilising the clinical record, ANZPICR, paper CRF)
- Data verification on the data items related to the calculation of the primary and secondary outcomes for every enrolled patient (utilising the clinical record, ANZPICR, paper CRF, intubation video)
- Data verification on key data items relating to cohort descriptors on a random sample of 10% of enrolled patients from each site (utilising the clinical record, ANZPICR)
- Data verification on reported adverse events for every enrolled patient (utilising the clinical record, intubation video)
- Clinical record and intubation video review for every enrolled patient to determine if all adverse events and protocol deviations have been reported

The original study REDCap database was enhanced to facilitate the activities outlined in the data monitoring plan. Each site is being monitored by a research coordinator not involved with the recruitment at that site. This is being undertaken through a combination of on-site monitoring and remote monitoring using the institutional programme to share desktop computer screens, remote access to electronic medical records as well as via the upload of source documents to the RDM. Uploaded videos are watched by the independent monitor and the relevant data items extracted and compared to those entered. During the coronavirus disease 2019 pandemic (COVID-19), monitoring is primarily being undertaken using remote methods; however, on-site monitoring may still occur following the easing of COVID-19 restrictions. Each relevant data item is verified individually by comparing the entered value with the value in the source documentation. Where discrepancies are found, the site research coordinator and monitor meet to discuss and resolve the discrepancies. Once data monitoring is finalised, the patient’s REDCap data entry record is locked in preparation for analysis.

### Statistical principles

The primary analysis will be conducted based on the intention-to-treat principle. Specifically, each randomised intubation encounter will be analysed based on the allocated treatment group, independent of compliance with the treatment delivered.

A per-protocol analysis including all intubation attempts where a study therapy was commenced (regardless of whether it was the therapy that was randomised) will also be undertaken and reported in the supplementary material.

The unit of analysis for most outcomes is the intubation encounter. If patients are required to be intubated more than once, they can be re-enrolled and re-randomised, theoretically receiving different study interventions for each intubation attempt within a single admission. For secondary outcomes relating to admission (i.e. ventilator-free days, length of ICU and hospital stay and death), only those patients with one enrolment per admission will be included in the primary and per-protocol analyses.

Statistical tests will be two-sided applying a statistical significance level of 0.05, although the primary outcomes will be analysed at a level of 0.025 (applying Bonferroni’s correction). We will not apply a formal correction for multiple testing to any of the subgroup analyses, sensitivity analyses or to the secondary outcomes. We will ensure conclusions drawn as a result of analyses are interpreted with deference to multiple comparisons.

If there is more than 10% of data missing for the primary outcomes, a sensitivity analysis using multiple imputation will be undertaken.

Continuous variables will be assessed for normality; this will be undertaken using visual inspection of histograms and *Q*-*Q* plots.

Descriptive statistics will be used when summarising variables; frequencies (percentages) for discrete variables, mean and standard deviation (SD) for normally distributed continuous variables, or, if continuous variables are non-normally distributed, median with interquartile range (IQR).

Subgroup analyses will be executed regardless of any potential treatment effect on the primary or secondary outcomes in the main cohort.

Changes to the analysis plan by the investigators effective after the publication of this SAP will be declared as such.

The statistical analysis will be undertaken using StataSE version 17 (StataCorp Pty Ltd., College Station, TX).

### Trial population

#### Screening data

**Fig. 1:**
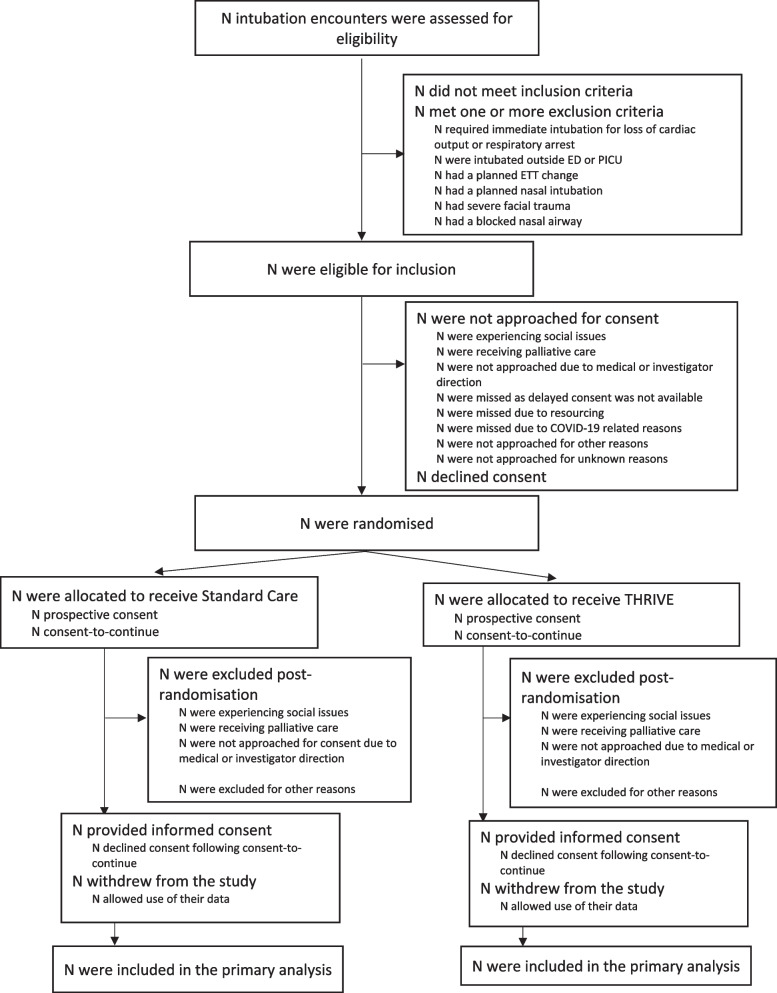
Proposed CONSORT participant flow diagram

All intubation encounters at participating sites will be assessed for eligibility for inclusion in the trial. The planned Consolidated Standards of Reporting Trials (CONSORT) [8] flow diagram will include all patients being screened for the study (Fig. 1). This will describe screened patients, those meeting exclusion criteria, eligible patients, consent process, those randomised into each of the study arms, with the documentation of the primary outcome. We will report on the start and stop date of the trial and provide the recruitment graph by month including division into the contributing sites as a supplementary figure.

#### Eligibility

Any patient undergoing endotracheal intubation in a participating department is eligible for inclusion. The specific inclusion and exclusion criteria are detailed in the protocol [4].

#### Withdrawal

If consent is not obtained or is withdrawn, data will be excluded from the analyses, unless permission is granted to use data recorded prior to the point of study withdrawal.

#### Baseline patient characteristics

Baseline characteristics (including demographic data, comorbidities, previous intubation details) at the time of randomisation will be reported for each of the two treatment groups (statistical comparison between groups will not be undertaken) (Table 1). Heart rate, respiratory rate and blood pressure will be presented as centiles, according to published charts [9, 10]. A supplementary table will be provided detailing additional baseline characteristics related specifically to the neonatal population.

**Table 1:** Demographic and pre-intubation surgical characteristics of participants enrolled in the Kids THRIVE trial

Characteristic	Standard care, *N* =	THRIVE, *N* =
**Age at randomisation (years), ** *mean (SD)/median (IQR)*		
**Age at randomisation (years)** ^**a**^ **,** *n (%)*		
1 year		
1–7 years		
> 7 years		
**Weight (kg),** *mean (SD)/median (IQR)*		
**Female sex,** *n (%)*		
**Ethnicity,** *n (%)*		
Caucasian		
Aboriginal/Torres Strait Islander		
Asian		
African		
Indian		
Middle Eastern		
Māori		
Pacific Islander		
Others		
Unknown		
**Premature at birth (< 37 weeks),** *n (%)*		
**Intracardiac right-to-left shunt present at the time of intubation,** *n (%)*		
**Previous intubation,** *n (%)*		
**Cormack-Lehane laryngeal grade**		
Grade 1,* n (%)*		
Grade 2,* n (%)*		
Grade 3,* n (%)*		
Grade 4,* n (%)*		

*SD* standard deviation, *IQR* interquartile range, *MgSO*_*4*_ magnesium sulphate

^a^Used for stratification

### Analysis

#### Outcome definitions

*Intubation attempt* is defined as a single advanced airway manoeuvre beginning with the insertion of the laryngoscope into the child’s mouth and ending when the laryngoscope is removed from the child’s mouth, or where there is a change in operator during the procedure even if the device is not removed.

*Hypoxaemia* is defined as transcutaneous oxygen saturations (SpO_2_) of ≤ 90% or a SpO_2_ saturation difference ≥ 10% for patients with cyanotic congenital heart disease with known substantial right-to-left shunts measured with the bedside monitor and with an accurate quality of the trace during the intubation attempt. Oxygen saturations will continue to be recorded for at least 2 min after the intubation attempt.

A *successful first attempt intubation* is defined as a successful placement of an endotracheal tube at first attempt, without any hypoxemia (SpO_2_ ≤ 90%or saturation difference ≥ 10% for right-to-left shunt). An unsuccessful first attempt intubation is either a successful first attempt intubation associated with hypoxemia, or requirement for more than one intubation attempt.

*Intubation attempt with rescue oxygenation* is defined as the provision of rescue oxygenation (for example, by bag and mask system) following an unsuccessful intubation attempt.

The following are the primary outcomes:

The clinically relevant and patient-centred outcome measures for intubation are hypoxemia and the number of attempts to achieve successful endotracheal intubation, both of which are strongly linked. Therefore, this study has two primary outcomes:

1. Intubation attempt without hypoxaemia
2. Successful first attempt intubation

The following are the secondary outcomes:

- Number of intubation attempts
- Number of intubation attempts with rescue oxygenation
- Lowest oxygen saturations during each attempt *(intubation attempt as unit of analysis)*
- Lowest oxygen saturation in the 2 min immediately following an intubation attempt
- Lowest oxygen saturation throughout total intubation encounter (defined as the first insertion of laryngoscope to successful intubation)
- Duration of invasive mechanical ventilation, defined as the start of successful intubation to extubation
- Ventilation-free days (VFD), defined as the duration of respiratory support for all episodes with an ETT in situ for the first 28 days after randomisation censored at 28 days; VFD will be recorded as 0 in patients that died within 28 days after randomisation
- Length of ICU stay measured in days, defined as the start of successful intubation to ICU discharge, and limited to patients with an ICU admission
- Length of hospital stay measured in days, defined as the start of successful intubation to hospital discharge
- Occurrence of minor tracheal intubation-related adverse events (TIAEs) defined as one of the following in the period starting at the commencement of the intubation attempt until 2 min after successful intubation:
  - Bradycardia, not requiring treatment
  - Hypotension, not requiring treatment
  - Main stem bronchial intubation
  - Oesophageal intubation with immediate recognition
  - Emesis without aspiration
  - Epistaxis
  - Dental or lip trauma
- Occurrence of major TIAEs defined as one of the following in the period starting at the commencement of the intubation attempt until 2 min after successful intubation:
  - Cardiac arrest with or without return of spontaneous circulation
  - Oesophageal intubation with delayed recognition (> 60 s)
  - Emesis with aspiration
  - Hypotension requiring treatment
  - Bradycardia requiring treatment
  - Laryngospasm
  - Malignant hyperthermia
  - Pneumothorax or pneumomediastinum
- Death, defined as death during current hospital admission

#### Analysis methods

##### Primary outcome measures

The primary outcome measures will be analysed using logistic regression adjusting for the treatment group and the stratification variables (age group, operator level) as fixed effects and site and patient as random effects (allowing for the same patient to be recruited more than once into the study). A model without adjustment for age group or operator level will also be reported. Unadjusted and adjusted odds ratios (ORs), 95% confidence intervals (CIs) and *p*-values for the adjusted model will be reported (Table 2). Assumptions of the models will be tested and reported on.

**Table 2:** Intubation characteristics of participants enrolled in the Kids THRIVE trial

Characteristic	Standard care, *N* =	THRIVE, *N* =
**Intubation location,** *n (%)*		
Emergency department		
Paediatric intensive care		
Neonatal intensive care		
Others		
**Reason for ED presentation/ICU admission**		
Sepsis,* n (%)*		
Trauma,* n (%)*		
Respiratory distress,* n (%)*		
Respiratory distress syndrome (RDS)/hyaline membrane disease (HMD),* n (%)*		
Meconium aspiration syndrome (MAS),* n (%)*		
Apnoea,* n (%)*		
Airway obstruction,* n (%)*		
Altered level of consciousness,* n (%)*		
Seizures,* n (%)*		
Overdose/intoxication,* n (%)*		
Congenital heart disease,* n (%)*		
Heart failure,* n (%)*		
Congenital abnormality,* n (%)*		
Malignancy,* n (%)*		
Pre-operative ICU admission,* n (%)*		
Post-operative ICU admission,* n (%)*		
Burns,* n (%)*		
Others,* n (%)*		
**Indication for intubation**		
Inadequate oxygenation,* n (%)*		
Inadequate ventilation,* n (%)*		
Unprotected airway,* n (%)*		
Haemodynamic instability,* n (%)*		
Procedure/intervention required,* n (%)*		
Expected clinical deterioration,* n (%)*		
Failed extubation,* n (%)*		
Upper airway obstruction,* n (%)*		
Others,* n (%)*		
**Pre-intubation observations**		
SpO_2_ * mean (SD)/median (IQR)*		
Hypotension, *n (%)*		
Heart rate centile		
< 10th centile,* n (%)*		
10–90th centile,* n (%)*		
> 90th centile,* n (%)*		
Respiratory rate centile		
< 10th centile,* n (%)*		
10–90th centile, *n (%)*		
> 90th centile,* n (%)*		
**Respiratory support, prior to intubation**		
Room air/nil support,* n (%)*		
Standard oxygen therapy, *n (%)*		
Nasal high flow, *n (%)*		
Bag-mask system,* n (%)*		
Non-invasive ventilation,* n (%)*		
Laryngeal mask airway, *n (%)*		
**Pre-oxygenation technique**		
Room air/nil support,* n (%)*		
Standard oxygen therapy, *n (%)*		
Nasal high flow, *n (%)*		
Bag-mask system,* n (%)*		
Non-invasive ventilation,* n (%)*		
Laryngeal mask airway, *n (%)*		
**Operator level for first intubation attempt** ^**a**^ **,** * n (%)*		
Consultant		
Fellow or registrar		

*SD* standard deviation, *IQR* interquartile range, *ED* emergency department, *ICU* intensive care unit, *ET* endotracheal tube

^a^Used for stratification

##### Secondary outcome measures

Binary outcome measures (e.g. occurrence of AE) will be treated in the same manner as the primary outcome; however, no *p*-values will be reported (Table 3). Similar analyses will be undertaken for continuous outcomes: linear regression with adjustment for the treatment group and stratification variables as fixed effects and site and patient as random effects, with reporting of mean difference (unadjusted for stratification variable and adjusted) and 95% CIs. If necessary, quantile regression will replace linear regression in the instance of highly skewed data. Survival outcomes (length of PICU stay, length of hospital stay) will be visually presented using a Kaplan–Meier plot, and a Cox proportional hazard model will be used to assess the differences between the treatment groups with treatment group and stratification variables as fixed effects and site and patient as random effects (i.e. utilising a shared frailty model). The proportionality assumption will be inspected visually using Kaplan–Meier plots and the log–log plot. The hazard ratio and 95% CI will be presented as an estimate of treatment effect. Competing-risk regression will be used to analyse VFDs (competing risk of mortality with duration of ventilation). The lowest oxygen saturation during each intubation encounter will be reported in-text using each encounter as the unit of analysis (and as such incorporating intubation episode as a further random effect into the model). Key assumptions of the models (for logistic regression: specification, goodness-of-fit, absence of multicollinearity and absence of influential observations; for survival analysis: proportionality assumption, goodness-of-fit; for linear regression: specification, distribution of residuals, homoscedasticity, absence of multicollinearity, linearity) will be tested and reported on.

**Table 3:** Primary outcomes in the total trial cohort and subgroups as per intention-to-treat analysis

	**Standard care, ** ***N*** ** = **	**THRIVE, = **	Estimate of difference (95% CI)*	**Adjusted estimate of difference (95% CI)** ^**a**^	***p*** **-value**
**Outcome: hypoxic event** *Oxygen saturations (SpO*_*2*_ *) of* ≤ *90% or a SpO*_*2*_ *saturation difference* ≥ *10% for patients with cyanotic congenital heart disease with known substantial right-to-left shunts*					
**Total trial cohort,** * n (%)*					
**Subgroup: patient age^**					
< 1 year,* n (%)*					
1–7 years,* n (%)*					
> 7 years,* n (%)*					
**Subgroup: operator level^**					
Junior, *n (%)*					
Senior,* n (%)*					
**Subgroup: location of intubation^**					
Emergency department,* n (%)*					
Paediatric Intensive Care Unit,* n (%)*					
Neonatal intensive care unit,* n (%)*					
**Outcome: successful first attempt intubation** *First attempt without any hypoxemia (SpO*_*2*_ ≤ *90% or saturation difference* ≥ *10% for right-to-left shunt)*					
**Total trial cohort,** * n (%)* ^a,^*					
**Subgroup: patient age^**					
< 1 year,* n (%)*					
1–7 years,* n (%)*					
> 7 years,* n (%)*					
**Subgroup: operator level^**					
Junior, *n (%)*					
Senior,* n (%)*					
**Subgroup: location of intubation^**					
Emergency department,* n (%)*					
Paediatric intensive care unit,* n (%)*					
Neonatal intensive care unit,* n (%)*					

*IQR* interquartile range, *CI* confidence interval

^*^Adjusted for study site and patient only; *p*-value = xx

^a^Adjusted for age at randomisation, operator level, study site and patient

^*p*-value represents interaction term

We will undertake the following subgroup analyses (Table 2):

1. Age group at randomisation (stratification variable; pre-planned)
2. Operator level (stratification variable; pre-planned)
3. Location of intubation (ED, PICU or NICU; defined after commencement of trial)

All subgroup analyses will examine the primary outcome only. Subgroup analyses will be undertaken using the same analysis methods described for the primary outcome measure, with the addition of the subgroup variable and its related interaction term into the main regression model; the interaction effect (and 95% CI and *p*-value) will be reported, alongside the descriptive statistics for the outcome under investigation. A forest plot will be developed to present heterogeneity between the treatment group and subgroup variable, including the *p*-value, and presented. Additionally, a sensitivity analysis will be undertaken based on the lowest SpO_2_ during intubation (< 80%; ≥ 80%).

#### Missing data

The primary outcome for this study is recorded by two methods, paper CRF and video recording, as such it is expected that there will be very few participants with missing primary outcome data. Where the data is not recorded on either the paper CRF or video, the medical record will be used to source the required data. As such, multiple imputation will only be undertaken if > 10% of the primary outcome data is unavailable and reported as sensitivity analysis. The fully conditional specification will be used for imputation; the imputation model will include randomised treatment arm, study site and the stratification variable. Ten sets of imputed data will be created using the methods described for the primary outcome. A pooled common effect estimate and 95% confidence interval will be generated from the imputed datasets.

#### Additional analyses

Analyses that have not been pre-defined in the protocol may be performed if requested by journal editors or reviewers. Any such analysis will be performed in a manner consistent with this analysis plan where possible. Subsequent exploratory analyses of the dataset following the publication of the main outcomes paper will not be bound by this strategy but will follow the broad principles we describe here.

#### Adverse events and protocol deviations

All adverse events defined in the study protocol will be reported as described in Table 3 and compared between the two study groups using logistic regression as described above for secondary outcomes (Table 4). Protocol deviations will be tabulated and reported in the supplementary material.

**Table 4:** Secondary outcomes in the total trial cohort

Outcome	Standard care, *N* =	THRIVE, *N* =	Estimate of difference (95% CI)^a^	Adjusted estimate of difference (95% CI)^b^
Number of intubation attempts, *median (IQR)*				
Number of intubation attempts with rescue oxygenation, *median (IQR)*				
Lowest oxygen saturation throughout total intubation period, *median (IQR)*				
Duration of mechanical ventilation, *median (IQR)*				
Ventilation free days, *median (IQR)* ^c^				
Length of ICU stay (days), *median (IQR)* ^c,^ ^d^				
Length of hospital stay (days), *median (IQR)* ^c^				
Minor adverse events				
Bradycardia, not requiring treatment,* n (%)*				
Hypotension, not requiring treatment,* n (%)*				
Main stem bronchial intubation,* n (%)*				
Oesophageal intubation with immediate recognition,* n (%)*				
Emesis without aspiration,* n (%)*				
Epistaxis,* n (%)*				
Dental or lip trauma,* n (%)*				
Major adverse events				
Cardiac arrest with or without return of spontaneous circulation,* n (%)*				
Oesophageal intubation with delayed recognition (> 60 s),* n (%)*				
Emesis with aspiration,* n (%)*				
Hypotension requiring treatment,* n (%)*				
Bradycardia requiring treatment,* n (%)*				
Laryngospasm,* n (%)*				
Malignant hyperthermia,* n (%)*				
Pneumothorax or pneumomediastinum,* n (%)*				
Death during current hospital admission, *n (%)* ^c^				

*SD* standard deviation, *IQR* interquartile range, *CI* confidence interval

^a^Adjusted for study site only

^b^Adjusted for age at randomisation, operator level and study site

^c^Limited to those patients with one enrolment per admission or multiple enrolments receiving the same randomised treatment

^d^Patients with an ICU admission only

## Administrative information

### Trial title and registration

Transnasal humidified rapid insufflation ventilatory exchange in children requiring emergent intubation (Kids THRIVE). This trial is registered in the Australian New Zealand Clinical Trial Registry (ANZCTR12617000147381).

### Protocol versio﻿n

This SAP is based on version 11 of the study protocol. Minor modifications to the original study protocol were reviewed and approved by the HREC and are detailed in the Supplementary Information (Additional file 1).

### SAP version

Version 1 (dated 6 August 2022).

### Roles and responsibilities

#### Trial Steering Committee

A/Prof Shane George (Chair)

A/Prof Susan Humphreys

Dr Ben Gelbart

Prof Andreas Schibler

#### Trial Statistician

A/Prof Kristen Gibbons

#### Study Co-ordinator

Ms Tara Williams

#### The Kids THRIVE investigators

Shane George, Joanna Cronin (Gold Coast University Hospital); Susan Humphreys, Tara Williams, Kylie Pearson, Katie Rasmussen, Jason Acworth (Queensland Children’s Hospital); Ben Gelbart, David Tingay, Leah Hickey, Carmel Delzoppo, Elizabeth Perkins (Royal Children’s Hospital Melbourne); Felix Oberender, Simon Craig, Jessica Waghorn (Monash Children’s Hospital); Arjun Chavan, Courtney McCahill (Townsville University Hospital); Nitesh Singhal, Gail Harper (Westmead Children’s Hospital); Subodh Ganu, Georiga Letton (Women’s and Children’s Hospital Adelaide); Simon Erikson, Hannah Thomson (Perth Children’s Hospital); Luregn J Schlapbach, Juerg Burren, Elisa Zimmerman (Department of Intensive Care and Neonatology, and Children`s Research Center, University Children’s Hospital Zurich, Zurich, Switzerland); Anusha Ganeshalingham, Stuart Dalziel, Clarie Sherring (Starship Children’s Hospital); Andreas Schibler (Wesley Medical Research).

#### Data and Safety Monitoring Board

A/Prof Paula Lister (Chair, Paediatric Intensivist)

Dr Heinrich Cornelissen (Paediatric Anaesthetist)

Dr Chris Flatley (Statistician and Epidemiologist)

### Supplementary Information

**Additional file 1:** **S1.** List of approved protocol modifications.

## List of planned Appendix material

- Data and Safety Monitoring Board terms of reference/charter
- Monitoring plan
- Enrolment statistics by month, site, and country
- Results of interim analyses
- Neonatal characteristics of participants enrolled in at a neonatal intensive care unit site or aged < 28 days at time of intubation
- Kaplan–Meier survival curves for length of PICU stay and length of hospital stay
- Forest plot of the treatment effect across subgroups for the primary and secondary outcomes
- Results of per-protocol analysis
- Results of sensitivity analysis
- List of protocol deviations
- List of adverse events

## References

[1] Lee JH, Turner DA, Kamat P, Nett S, Shults J, Nadkarni VM, Nishisaki A, Pediatric Acute Lung, I., Sepsis, I., and National Emergency Airway Registry for, C (2016). The number of tracheal intubation attempts matters! A prospective multi-institutional pediatric observational study. BMC Pediatr..

[2] Weingart SD, Levitan RM (2012). Preoxygenation and prevention of desaturation during emergency airway management. Ann Emerg Med.

[3] Humphreys S, Lee-Archer P, Reyne G, Long D, Williams T, Schibler A (2017). Transnasal humidified rapid-insufflation ventilatory exchange (THRIVE) in children: a randomized controlled trial. Br J Anaesth.

[4] George S, Humphreys S, Williams T, Gelbart B, Chavan A, Rasmussen K, Ganeshalingham A, Erickson S, Ganu S, Singhal N, Foster K, Gannon B, Gibbons K, Schlapbach L, Festa M, Dalziel S, Schibler A (2019). Transnasal Humidified Rapidinsufflation Ventilatory Exchange in children requiring emergent intubation (Kids THRIVE): a protocol for a randomised controlled trial. BMJ Open.

[5] Harris PA, Taylor R, Minor BL, Elliott V, Fernandez M, O’Neal L, McLeod L, Delacqua G, Delacqua F, Kirby J, Duda SN, Consortium, R.E (2019). The REDCap consortium: building an international community of software platform partners. J Biomed Inform..

[6] Harris PA, Taylor R, Thielke R, Payne J, Gonzalez N, Conde JG (2009). Research Electronic Data Capture (REDCap)–a metadata-driven methodology and workflow process for providing translational research informatics support. J Biomed Inform.

[7] International Council for Harmonisation of Technical Requirements for Pharmaceuticals for Human Use. ICH Harmonised Guideline: Integrated Addendum to ICH E6 (R1): guideline for good clinical practice E6 (R2). 2016.

[8] Schulz KF, Altman DG, Moher D, Group, C (2010). CONSORT 2010 statement: updated guidelines for reporting parallel group randomised trials. BMJ..

[9] Bonafide CP, Brady PW, Keren R, Conway PH, Marsolo K, Daymont C (2013). Development of heart and respiratory rate percentile curves for hospitalized children. Pediatrics.

[10] National Heart L, Blood Institute. Diagnosis, evaluation, and treatment of high blood pressure in children and adolescents. Bethesday: National Institutes of Health, Editor; 2005.

