# Supplementary material for: Transnasal Humidified Rapid Insufflation Ventilatory Exchange in children requiring emergent intubation (Kids THRIVE): a statistical analysis plan for a randomised controlled trial
Source: Trials. 2023 May 31;24:369. doi: 10.1186/s13063-023-07330-z (PMC10230452; doi:10.1186/s13063-023-07330-z)
Supplement: Supplementary file 1 — Additional file 1: S1. List of approved protocol modifications. [file 13063_2023_7330_MOESM1_ESM.docx]

**Supplementary Appendix**

**S1. List of approved protocol modifications**

| **Version Number and Date** | **Ethics Committee Approval Date** | **List of Modifications** |
| --- | --- | --- |
| ​Version 1.0  ​20 November 2016 | NA | ​First version |
| ​Version 2.0  ​15 December 2016 | 20/12/2016 | - ​Minor changes as requested by HREC |
| ​Version 3.0  ​6 March 2017 | 29/03/2017 | - ​Addition of video recording as data collection method.  Consent process modified to use wording “Consent to Continue”. Minor grammatical and typographical corrections. |
| ​Version 4.0  ​17 April 2017 | 28/04/2017 | - ​Change of video data storage terms. The addition of consideration to be given to children where 100% oxygen is contraindicated. |
| ​Version 5.0  ​1 November 2017 | 16/11/2017 | - ​Nasal intubation added to exclusion criteria. Dr Gelbart added as CI. Clarification of video collection and adverse event definitions. |
| ​Version 6.0  ​8 May 2018 | 31/05/2018 | - ​Clarification of Primary and Secondary outcome measures, statistical analysis, and sample size calculations. Addition of Health Economic evaluation details. Addition of stratification by operator. |
| ​Version 7.0  ​1 November 2018 | 22/11/2018 | - ​Further clarification for ease for understanding in multiple sections of the protocol based on extensive peer review. Addition of new sites and changes to investigators. |
| ​Version 8.0  ​13 May 2019 | 27/05/2019 | - ​Revision to expand to international sites, minor wording changes and addition of sites and investigators. Updated DSMB review points. |
| ​Version 9.0  ​1 December 2019 | 08/01/2020 | - ​Revision to expand to NICU and the addition of sites and investigators |
| ​Version 10.0  ​1 July 2020 | 20/07/2020 | - ​Inclusion of ANZPIC Registry as data source, updated sites, and investigators |
| Version 11  11 July 2020 | 03/08/2020 | - COVID-19 pandemic verbal consent |
